# Isolation of human ESC-derived cardiac derivatives and embryonic heart cells for population and single-cell RNA-seq analysis

**DOI:** 10.1016/j.xpro.2021.100339

**Published:** 2021-02-11

**Authors:** Federica Santoro, Kenneth R. Chien, Makoto Sahara

**Affiliations:** 1 Department of Cell and Molecular Biology, Karolinska Institutet, Stockholm 171 77, Sweden; 2 Department of Medicine, Karolinska Institutet, Stockholm 141 86, Sweden; 3 Institution of Immunology, Genetics and Pathology, Uppsala Universitet, Uppsala 751 85, Sweden; 4 Department of Surgery, Yale University School of Medicine, New Haven, CT 06510, USA

**Keywords:** Cell differentiation, Cell isolation, Flow cytometry/mass cytometry, RNA-seq, Single cell, Stem cells

## Abstract

The combination of population and single-cell RNA sequencing analysis using human embryonic stem cell (hESC) differentiation and developmental tissues is a powerful approach to elucidate an organ-specific cellular and molecular atlas in human embryogenesis. This protocol describes (1) cardiac-directed differentiation and isolation of hESC-derived cardiac derivatives with fluorescence-activated cell sorting, (2) isolation of human embryonic heart-derived single cardiac cells, and (3) construction of cDNA libraries with Smart-seq2. These allow for the preparation of human developmental samples for comprehensive transcriptional analysis.

For complete details on the use and execution of this protocol, please refer to Sahara et al. (2019).

## Highlights

• Efficient cardiac-directed differentiation of hESCs
• Isolation of hESC-derived cardiac derivatives with fluorescence-activated cell sorting
• Isolation of human embryonic heart-derived single cardiac cells
• Construction of cDNA libraries for both population and single-cell RNA sequencing

## Graphical abstract

**Figure:**
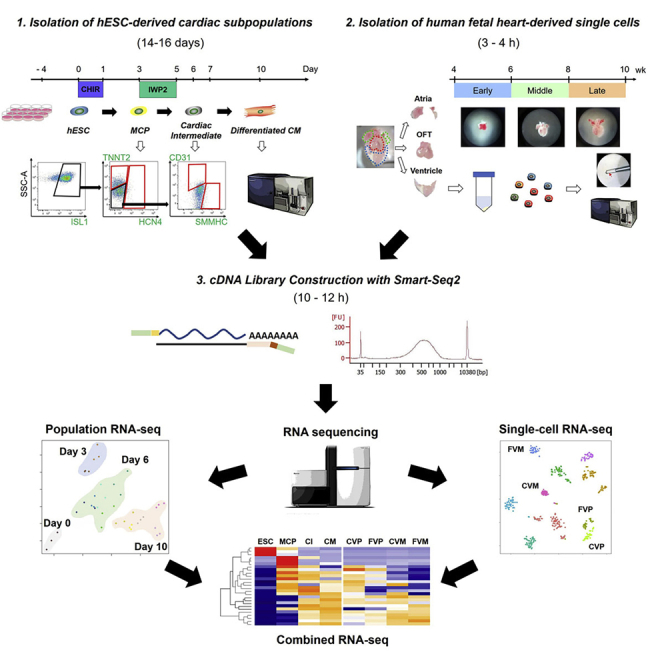

## Before you begin

### Maintenance of hESC culture

1. Prepare Matrigel-coated 6-well and 12-well plates:
  a. Thaw frozen Matrigel (BD Biosciences) at 4°C for 12–16 h. Aliquot and freeze again directly.
    ***Note:*** Store the aliquots at −20°C for up to 6 months. The use of pre-cooled pipets, tips, and tubes are recommended when handling Matrigel, to avoid forming a gel.
  b. Resuspend thawed Matrigel in cold DMEM/F12 at a 1:100 ratio (e.g., 120 μL of Matrigel in 12 mL of DMEM/F12).
  c. Add 1 mL (or 0.5 mL) of diluted Matrigel per well of a 6-well (or 12-well) plate (approximately 7–10 μg/cm^2^). Ensure that the entire surface in each well is covered.
  d. Store the coated plates at 4°C for up to 2 weeks.
  e. Before use, incubate the coated plate at 37°C for 30–45 min. Then, after aspirating Matrigel solutions, the plate is ready to seed fresh hESCs for cell maintenance (on 6-well plates) or differentiation (on 12-well plates).

2. Culture and maintain the human ESC lines (e.g., ES03) on Matrigel-coated 6-well plates in a humidified incubator at 37°C and 5% CO_2_. Cells are fed daily with mTeSR-1 medium and passaged every 5–7 days by incubating with Dispase (for 10–15 min at 37°C) or Accutase (for 5–10 min at 37°C). For the first 24 h after subculturing, the media should be supplemented with 5 μM Y-27632 (a ROCK inhibitor).

***Alternatives:*** The Essential 8 medium and vitronectin (diluted at 1:100 in PBS)-coated plates, can be used as substitutes for mTeSR-1 medium and Matrigel-coated plates for maintenance culture of hESCs. When using the Essential 8 and vitronectin system, Versene solution (for 5–10 min at 37°C) is recommended to use as a non-enzymatic cell dissociation reagent for passaging the cells.

3. Before starting differentiation experiments, check if the cultured hESCs entirely express pluripotency markers (e.g., Oct3/4) via immunocytochemistry or flow cytometry, and verify that the hESCs show a normal karyotype using standard G-band karyotype analysis (Tidball et al., 2017; Sun et al., 2020).

### Human embryonic/fetal heart experiments

4. For human embryonic/fetal heart experiments, an Institutional Review Board (IRB)-approved ethical permission and approved informed consents must be first acquired. Human embryonic/fetal heart samples (e.g., 4.5 to 10 weeks of the gestation stages) should be obtained from authorized sources using an IRB-approved protocol (Sahara et al., 2019).

**CRITICAL:** Human samples are considered to be potentially infectious and should be handled in Biosafety Level II cabinets.

## Key resources table

**Table:** 

REAGENT or RESOURCE	SOURCE	IDENTIFIER
**Antibodies**		

See Table 1 for antibodies for flow cytometry	This paper	Table 1

**Biological samples**		

Human embryonic/fetal heart tissues	Authorized sources in Karolinska University Hospital (Sweden)	N/A

**Chemicals, peptides, and recombinant proteins**		

mTeSR1	STEMCELL Technologies	Cat.# 05850
Essential 8 medium	Thermo Fisher Scientific	Cat.# A1517001
RPMI	Thermo Fisher Scientific	Cat.# 11875119
DMEM/F12	Thermo Fisher Scientific	Cat.# 11320033
DPBS (1×) (no calcium, no magnesium)	Thermo Fisher Scientific	Cat.# 14190144
HEPES (1 M)	Gibco	Cat.# 11560496
Matrigel	BD Biosciences	Cat.# 354234
Vitronectin	Thermo Fisher	Cat.# A14700
Dispase	STEMCELL Technologies	Cat.# 07913
Accutase	STEMCELL Technologies	Cat.# 07920
Versene solution	Gibco	Cat.# 15040066
Y-27632	Tocris	Cat.# 1254
CHIR99021	Sigma-Aldrich	Cat.# SML1046
CHIR98014	Sigma-Aldrich	Cat.# SML1094
IWP-2	Tocris	Cat.# 3533
Wnt-C59	Tocris	Cat.# 5148
B-27 Supplement (50×)	Thermo Fisher Scientific	Cat.# 17504044
B-27 Supplement (50×), minus insulin	Thermo Fisher Scientific	Cat.# A1895601
DMSO	Sigma-Aldrich	Cat.# 2650
Hydrochloric acid (37%)	Sigma-Aldrich	Cat.# 320331
Saponin	Sigma-Aldrich	Cat.# 84510
Horse serum	Thermo Fisher Scientific	Cat.# 1605130
Bovine serum albumin	Sigma-Aldrich	Cat.# A2153
Paraformaldehyde	Sigma-Aldrich	Cat.# 158127
RNaseOUT ribonuclease inhibitor (40 U/μL)	Thermo Fisher Scientific	Cat.# 10777019
Collagenase type 2	Worthington	Cat.# 4176
Protease type XXIV	Sigma-Aldrich	Cat.# P8038
Fetal bovine serum	Sigma-Aldrich	Cat.# 12103C
TrypLE Express enzyme	Thermo Fisher Scientific	Cat.# 12604013
Triton X-100	Sigma-Aldrich	Cat.# X100
dNTP mix	New England Biolabs	Cat.# N0447S
SuperScript II reverse transcriptase	Thermo Fisher Scientific	Cat.# 18064014
KAPA HiFi HotStart ReadyMix	KAPA Biosystems	Cat.# KK2601
AMPure XP beads	Beckman Coulter	Cat.# A63881
DAPI solution (1.0 mg/mL)	BD Pharmingen	Cat.# 564907
PI solution (100 μg/2 mL)	BD Pharmingen	Cat.# 5564463

**Critical commercial assays**		

RecoverAll Total Nucleic Acid Isolation Kit	Ambion	Cat.# AM1975
Smart-Seq2	(Picelli et al., 2013)	N/A
Agilent RNA 6000 Nano Kit	Agilent	Cat.# 5067-1511
Agilent RNA 6000 Nano Chips	Agilent	Cat.# 5067-1529
Agilent High-Sensitivity DNA Kit	Agilent	Cat.# 5067-4626
Agilent High-Sensitivity DNA Chips	Agilent	Cat.# 5067-4627
Nextera XT DNA Library Preparation Kit	Illumina	Cat.# FC-131-1024
Nextera XT Index Kit (24 or 96 indexes for 96 or 384 samples)	Illumina	Cat.# FC-131-1001, # FC-131-2001
Qubit High-Sensitivity DNA Kit	Thermo Fisher Scientific	Cat.# Q32854

**Deposited data**		

Population RNA-seq data	This paper	E-MTAB-7537
Single-cell RNA-seq data	This paper	PRJNA510181

**Experimental models: cell lines**		

Human ESC: ES03 line	WiCell	Cat.# ES03

**Oligonucleotides**		

oligo-dT primer: 5′-AAGCAGTGGTATCAACGCAGAGT ACT_30_VN-3′	This paper	N/A
Template-switching oligo: 5′-AAGCAGTGGTATCAACGCAGAGT ACATrGrG+G-3′ (∗rG: riboguanosine; +G: Locked Nucleic Acid-modified guanosine)	QIAGEN	N/A
IS-PCR primer: 5′-AAGCAGTGGTATCAACGC AGAGT-3′	This paper	N/A

**Software and algorithms**		

FACS Diva	BD Biosciences	RRID:SCR_001456
FlowJo	Tree Star	RRID:SCR_008520

**Other**		

TC20 automated cell counter	Bio-Rad	Cat.# 1450102
Fine small scissor	Fine Science Tools	No. 14090-09
Fine spring scissor	Fine Science Tools	No. 15000-10
Fine forceps	Dumont	No. 11251-20, 11251-35
Magnetic Stand-96	Ambion	Cat.# AM10027
FACSARIA III cell sorter	BD Biosciences	RRID:SCR_016695
Bioanalyzer instrument	Agilent	RRID:SCR_018043
Qubit fluorometer	Thermo Fisher Scientific	RRID:SCR_018095
Hiseq 2500 system	Illumina	RRID:SCR_016383

## Materials and equipment

**Table:** RPMI/B27-ins medium

Reagent	Final concentration	Amount
RPMI	–	490 mL
B-27 Supplement (50×), minus insulin	1×	10 mL
**Total**	–	**500 mL**

**Table:** RPMI/B27 medium

Reagent	Final concentration	Amount
RPMI	–	490 mL
B-27 Supplement (50×)	1×	10 mL
**Total**	–	**500 mL**

**Table:** Y-27632, 5 mM (stock)

Reagent	Final concentration	Amount
Y-27632	–	10 mg
Sterile DPBS	–	6.24 mL
**Total**	–	**6.24 mL**

***Note:*** Dissolve, aliquot, and store at −20°C for up to 6 months or at 4°C for up to 2 weeks. Add in a medium at a 1:1,000 ratio (working concentration: 5 μM).

**Table:** CHIR99021, 30 mM (stock)

Reagent	Final concentration	Amount
CHIR99021	–	25 mg
DMSO	–	1.79 mL
**Total**	–	**1.79 mL**

***Note:*** Dissolve, aliquot, and store at −20°C for up to 6 months or at 4°C for up to 2 weeks. Add in a medium at a 1:2,500 ratio (working concentration: 12 μM).

***Alternatives:*** Another GSK-3β inhibitor CHIR98014 can be used as a substitute for CHIR99021 and applied as follows: dissolve 5 mg of CHIR98014 (Sigma) in 2.06 mL of DMSO (stock concentration: 5 mM). Aliquot and store at −20°C for up to 6 months or at 4°C for up to 2 weeks. Add in a medium at a 1:5,000 ratio (working concentration: 1 μM).

**Table:** IWP2, 5 mM (stock)

Reagent	Final concentration	Amount
IWP2	–	10 mg
DMSO	–	4.29 mL
**Total**	–	**4.29 mL**

***Note:*** Dissolve by incubating the mixture at 37°C for 10 min. Aliquot and store at −20°C for up to 6 months or at 4°C for up to 2 weeks. Add in a medium at a 1:1,000 ratio (working concentration: 5 μM).

***Alternatives:*** Another Wnt inhibitor Wnt-C59 can be used as a substitute for IWP2 and applied as follows: dissolve 10 mg of Wnt-C59 (Tocris) in 2.64 mL of DMSO (stock concentration: 10 mM). Aliquot and store at −20°C for up to 6 months or at 4°C for up to 2 weeks. Add in a medium at a 1:5,000 ratio (working concentration: 2 μM).

**Table:** FACS buffer

Reagent	Final concentration	Amount
Bovine serum albumin (BSA)	1%	1 g
Fetal bovine serum (FBS)	3%	3 mL
PBS (1×)	–	97 mL
**Total**	–	**100 mL**

***Note:*** Dissolve and filter the above. Aliquot and store at −20°C for up to 6 months or at 4°C for up to 2 weeks.

**Table:** Blocking/staining solution A

Reagent	Final concentration	Amount
Horse (or goat) serum	10%	10 mL
BSA	1%	1 g
PBS (1×)	–	90 mL
**Total**	–	**100 mL**

***Note:*** Dissolve and filter the above. Aliquot and store at −20°C for up to 6 months or at 4°C for up to 2 weeks.

**Table:** Blocking/staining solution B

Reagent	Final concentration	Amount
Horse (or goat) serum	10%	10 mL
BSA	1%	1 g
Saponin	0.1%	100 μg
PBS (1×)	–	90 mL
**Total**	–	**100 mL**

***Note:*** Dissolve and filter the above. Aliquot and store at −20°C for up to 6 months or at 4°C for up to 2 weeks.

**Table:** 4% paraformaldehyde (PFA) solution

Reagent	Final concentration	Amount
PFA	4%	4 g
PBS (1×)	–	100 mL
**Total**	–	**100 mL**

***Note:*** Dissolve 4 g of PFA in 100 mL PBS using microwave. Adjust the solution pH to 7.4 with 1 N NaOH and 1 N HCl. Aliquot and store at −20°C for up to 6 months or at 4°C for up to 2 weeks.

**Table:** Permeabilization solution

Reagent	Final concentration	Amount
Triton X-100	0.1%	100 μL
PBS (1×)	–	99.9 mL
**Total**	–	**100 mL**

***Note:*** Filter, aliquot and store at −20°C for up to 6 months or at 4°C for up to 2 weeks.

**Table:** Heart-primary solution

Reagent	Final concentration	Amount
HEPES (1 M)	10 mM	5 mL
NaCl solution (5 M)	35 mM	3.5 mL
Glucose (D(+), MW = 180.16)	10 mM	0.901 g
Sucrose (MW = 342.30)	134 mM	22.93 g
Na_2_HPO_4_ (MW = 141.96)	16 mM	1.136 g
NaHCO_3_ (MW = 84.01)	25 mM	1.050 g
KCl (MW = 74.56)	7.75 mM	0.289 g
KH_2_PO_4_ (MW = 136.09)	1.18 mM	0.080 g
HCl (37%)	–	Up to pH 7.4
PBS (1×)	–	Up to 500 mL
**Total**	–	**500 mL**

***Note:*** Prepare and dissolve the above. Filter and store at 4°C for up to 3 months.

**Table:** 10% bovine serum albumin (BSA) solution

Reagent	Final concentration	Amount
BSA	10%	10 g
PBS (1×)	–	Up to 100 mL
**Total**	–	**100 mL**

***Note:*** Dissolve 100 mg/mL of BSA in PBS. Aliquot and store at −20°C for up to 6 months or at 4°C for up to 2 weeks.

**Table:** Collagenase A solution

Reagent	Final concentration	Amount
Collagenase type II	200 U/mL	4.2 mg
Protease type XXIV	6 U/mL	7.74 mg
10% BSA solution	0.5%	600 μL
Heart-primary solution	–	11.4 mL
**Total**	–	**12 mL**

***Note:*** Dissolve and filter the above in each experimental case. This solution cannot be stocked.

**Table:** Collagenase B solution

Reagent	Final concentration	Amount
Collagenase type II	400 U/mL	24 mg
Heart-primary solution	–	40 mL
**Total**	–	**40 mL**

***Note:*** Dissolve and filter the above in each experimental case. This solution cannot be stocked.

**Table:** Cell suspension buffer

Reagent	Final concentration	Amount
TrypLE Express enzyme	–	5 mL
DPBS (1×)	–	5 mL
**Total**	–	**10 mL**

**Table:** Cell lysis buffer

Reagent	Final concentration	Amount
Triton X-100	0.2%	2 μL
RNase inhibitor (40 U/μL)	2 U/μL	50 μL
Nuclease-free water	–	948 μL
**Total**	–	**1 mL**

**Table:** RT mix

Reagent	Final concentration (in 10 μL)	Amount
SuperScript II reverse transcriptase (200 U/μL)	10 U/μL	0.5 μL
RNase inhibitor (40 U/μL)	1 U/μL	0.25 μL
SuperScript II first-strand buffer (5×)	1×	2 μL
DTT (100 mM)	5 mM	0.5 μL
Betaine (5 M)	1 M	2 μL
MgCl_2_ (1 M)	6 mM	0.06 μL
Template-switching oligo (100 μM)	1 μM	0.10 μL
Nuclease-free water	–	0.29 μL
**Total**	–	**5.7 μL**

**Table:** PCR pre-amplification mix

Reagent	Final concentration	Amount
First-strand reaction (from step 39)	–	10 μL
KAPA HiFi HotStart Ready Mix (2×)	1×	12.5 μL
IS-PCR primer (10 μM)	0.1 μM	0.25 μL
Nuclease-free water	–	2.25 μL
**Total**	–	**25 μL**

**Table:** Tagmentation reaction solution

Reagent	Final concentration	Amount
DNA from PCR (from step 42l)	–	500 pg
Tagment DNA buffer (2×)	1×	10 μL
Tagment DNA enzyme mix	–	5 μL
Nuclease-free water	–	Up to 20 μL
**Total**	–	**20 μL**

***Note:*** A tagment DNA buffer (2×) and a tagment DNA enzyme mix are supplied in the Nextera XT DNA Library Preparation kit (Illumina).

**Table:** Enrichment PCR solution

Reagent	Final concentration	Amount
Tagmented DNA (from step 44d)	–	25 μL
Nextera PCR Master Mix (2×)	1×	15 μL
Index 1 primers (N7xx)	–	5 μL
Index 2 primers (N5xx)	–	5 μL
**Total**	–	**50 μL**

***Note:*** A Nextera PCR Mater Mix is supplied in the Nextera XT DNA Library Preparation kit (Illumina), and Index 1 and 2 primers are supplied in the Nextera XT Index kit (24 or 96 indexes for 96 or 384 samples).

## Step-by-step method details

### Cardiac-directed differentiation of hESCs

**Timing: 14–16 days**

**Figure 1:**
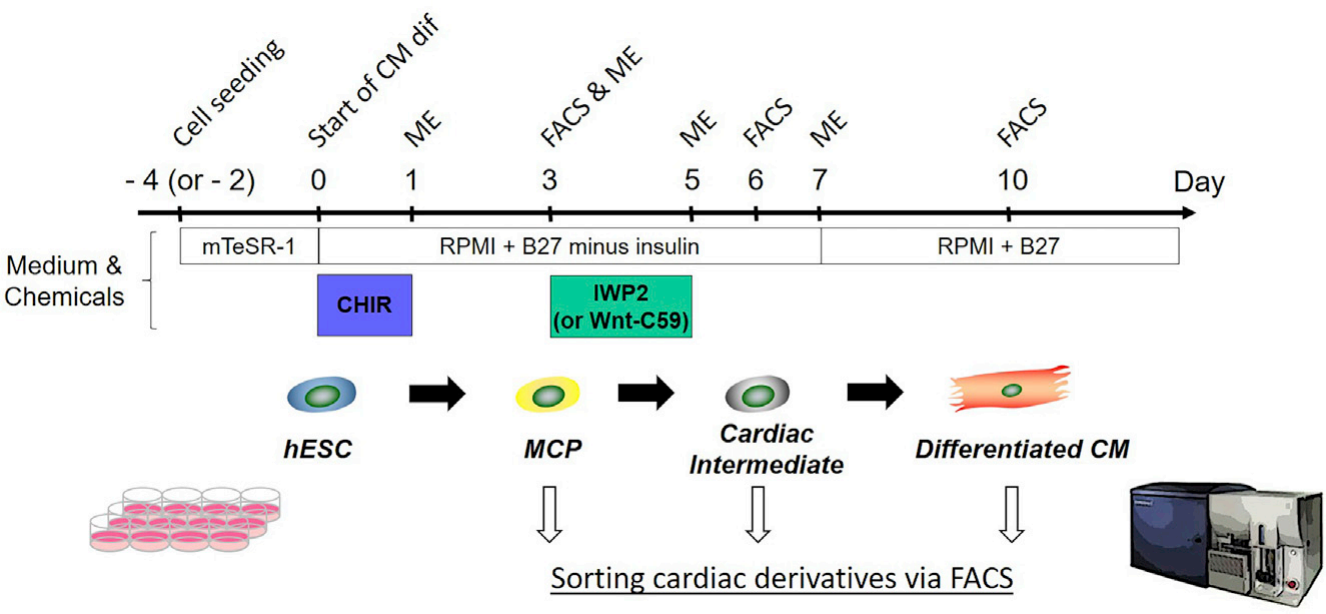
hESC cardiac differentiation protocol based on Wnt signaling modulation CM, cardiomyocyte; MCP, multipotent cardiac progenitor; ME, medium exchange.

Cardiac differentiation of hESCs is conducted with the Wnt signaling modulators, based on previously published protocols (Burridge et al., 2014; Lian et al., 2013) with minor modifications (Figure 1). This approach can generate efficiently cTnT^+^ cardiomyocytes occupying 60%–80% of the total differentiated cells in 10–12 days, via the ISL1^+^ multipotent cardiac progenitors (MCPs) and cardiac intermediates on day 3 and day 6 of differentiation, respectively. This protocol also generates small subsets of other cardiac derivatives, such as pacemaker, smooth muscle, and endothelial cells (Sahara et al., 2019).

**CRITICAL:** For efficient differentiation, it is highly recommended to use hESCs with less than 50–60 passages.

1. Four days before differentiation (day −4): Dissociate hESCs cultured on maintenance plates with 80%–90% confluency into single cells, and seed them on differentiation plates:
  a. Aspirate the old mTeSR1 medium on Matrigel-coated 6-well plates and wash the cells with DPBS once.
  b. Add 1 mL of Accutase to each well. Put the plate in a 37°C, 5% CO_2_ incubator for 5–10 min.
  c. Add 1 mL of mTeSR1 or fetal bovine serum (FBS) to each well. Collect all the cells into a 15-mL conical tube by gently pipetting.
    ***Note:*** Before use, warm all medium, serum, and solutions at 20°C–25°C for more than 20–30 min.
  d. Count the total cell number using an automated cell counter or a hemocytometer.
  e. Centrifuge the pooled cells at 200 × *g* for 5 min at 20°C–25°C.
  f. Aspirate the supernatant and resuspend the cells in mTeSR1 supplemented with 5 μM Y-27632 (a ROCK inhibitor) at an appropriate cell density (e.g., 0.5–1 million cells per mL).
  g. Seed 250,000–500,000 dissociated cells in a final volume of 1 mL media (mTeSR1 + 5 μM Y-27632) per well of Matrigel-coated 12-well plates.
    **CRITICAL:** The optimal seeding density on plates for efficient cardiac differentiation is variable and dependent on cell lines and passage numbers. The pilot experiments are required to determine it in each experimental case (see TroubleshootingProblem 1).

2. Days −3 to −1: The medium is replaced daily with 1 mL of fresh mTeSR1 per well of the 12-well plate.

***Alternatives:*** This initial cell growing period can be shortened from a total of 4 days to 2 days. If shortened, seed 750,000 to 1.2 million dissociated cells per well of Matrigel-coated 12-well plates in the beginning. The optimal seeding density should be determined empirically in each case.

3. Day 0: Aspirate the old mTeSR1 medium and add 2 mL of RPMI medium supplemented with B27 minus insulin (RPMI/B27-ins) and with 12 μM CHIR99021 (a GSK-3β inhibitor) per well of the 12-well plate.

***Note:*** The optimal concentration of CHIR99021 may vary a little depending on cell lines and other conditions, but it is usually 6–14 μM (see TroubleshootingProblem 1).

***Alternatives:*** 1 μM CHIR98014 can be used as a substitute for CHIR99021. The optimal concentration of CHIR98014 may vary a little depending on cell lines and other conditions but is usually 0.7–1.2 μM.

4. Day 1 (24 h after the addition of CHIR99021 on day 0): Aspirate the RPMI/B27-ins medium with CHIR99021 and add 2 mL of fresh RPMI/B27-ins medium per well of the 12-well plate.

**CRITICAL:** The RPMI/B27-ins medium with CHIR99021 should be replaced into fresh RPMI/B27-ins medium without CHIR99021 precisely after 24 h.

5. Day 3 (72 h after the addition of CHIR99021 on day 0): Prepare and add the semi-conditioned medium supplemented with IWP2 (a Wnt inhibitor):
  a. Collect 1 mL of the old medium per well of the 12-well plate into a 50-mL conical tube.
  b. Add 1 mL of fresh RPMI/B27-ins per well into the conical tube (Mix old and fresh RPMI/B27-ins medium at a 1:1 ratio).
  c. Add 2 μL of 5 mM IWP2 into 2 mL of the semi-conditioned medium from step 5b (final concentration 5 μM).
  d. Aspirate the remaining 1 mL of the old medium per well and add 2 mL of the semi-conditioned medium supplemented with IWP2.

***Alternatives:*** 2 μM (working concentration) of Wnt-C59 can be used as a substitute for 5 μM IWP2.

6. Day 5 (96 h after the addition of CHIR99021 on day 0): Aspirate the semi-conditioned medium with IWP2 and add 2 mL of fresh RPMI/B27-ins medium per well of the 12-well plate.
7. Day 7: Aspirate the old RPMI/B27-ins medium and add 2 mL of fresh RPMI medium supplemented with B27 (RPMI/B27) per well of the 12-well plate.
8. Thereafter, the medium is replaced into 2 mL of fresh RPMI/B27 per well of the 12-well plate every 2 or 3 days.
9. Beating cardiomyocytes start to appear in the culture, typically at day 8–9 of cardiac differentiation. Robust and broad spontaneous contractions are observed from day 10–12 onward.

### Isolation of hESC-derived cardiac subpopulations with FACS

**Timing: 5–6 h**

**Figure 2:**
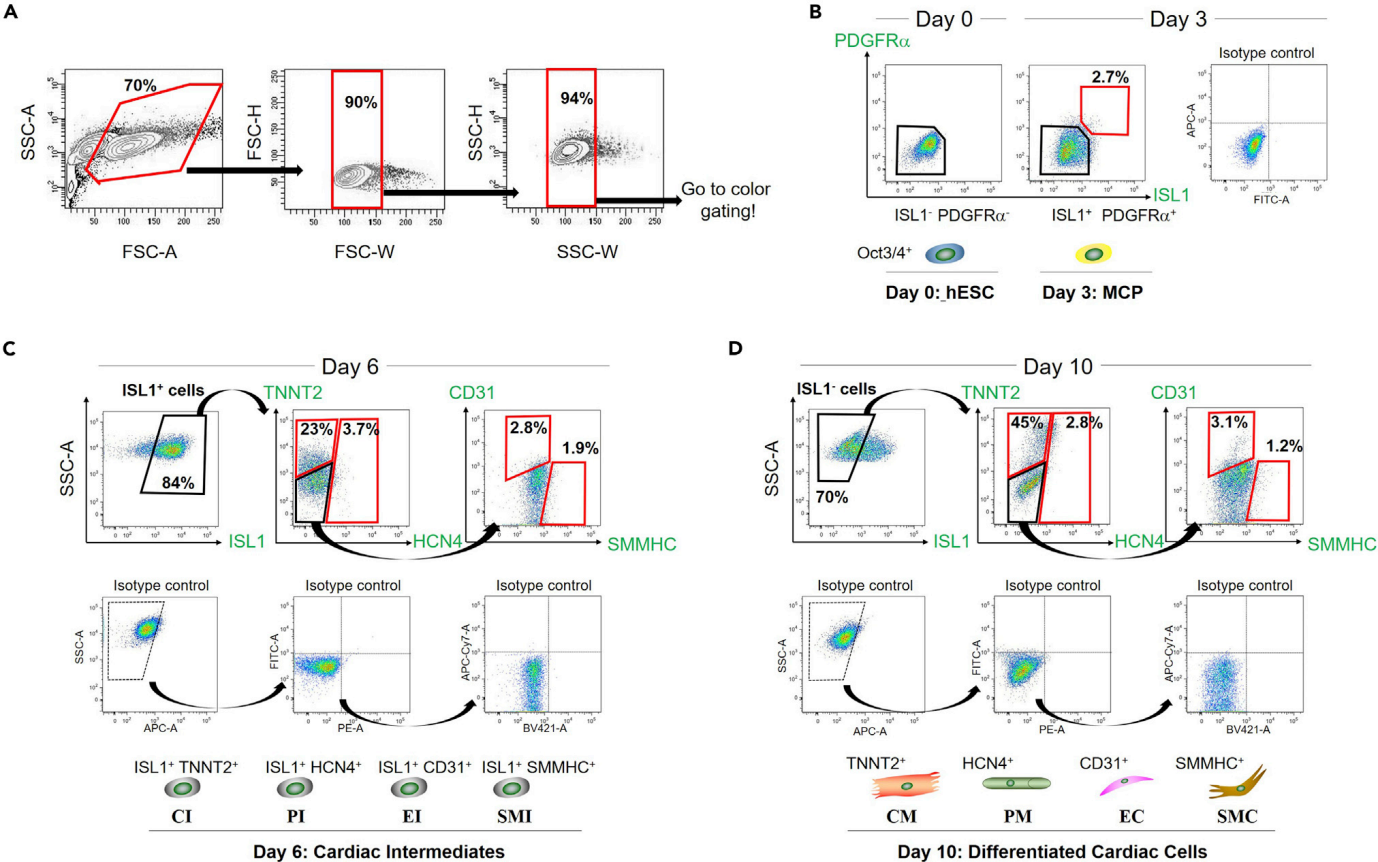
Gating strategies for sorting hESC-derived cardiac subpopulations Representative FACS plots showing examples of gating strategies for the isolation of hESC-derived cardiac derivatives. (A) The first gating of cells on the FSC and SSC plots to discard cell debris and doublets (each time). (B–D) Gating the ISL1^+^PDGFRα^+^ MCPs (day 3; B), the ISL1^+^Lin^+^ cardiac intermediates (day 6; C), and the ISL1^−^Lin^+^ differentiated cardiac cells (day 10; D). The plots of cells stained with isotype controls are also shown, respectively. CI, cardiomyocyte intermediate; CM, cardiomyocyte; EC, endothelial cell; EI, endothelial intermediate; MCP, multipotent cardiac progenitor; PI, pacemaker intermediate; PM, pacemaker cell; SMC, smooth muscle cell; SMI, smooth muscle intermediate.

Following the *in vitro* cardiac differentiation as noted above, a variety of hESC-derived cardiac subpopulations that involve early MCPs to cardiac intermediates and differentiated cardiac cells can be isolated at different time points. During differentiation, ISL1^+^ cardiac progenitors first appear on day 3 and peak by day 6 when ISL1^+^ cells occupy 80%–90% of the total differentiated cells. These cells mainly differentiate into beating cardiomyocytes along with generating small subsets of other differentiated cell types, such as pacemaker, smooth muscle, and endothelial cells from day 8–10 onward. The isolation of the hESC-derived cardiac subpopulations is conducted using fluorescence-activated cell sorting (FACS) and lineage-specific markers’ antibodies for cell staining on days 3 (for MCPs), 6 (for cardiac intermediates), and 10 (for differentiated cardiac cells) of the differentiation, respectively (Figures 1 and 2).

10. On each of the specified days (i.e., days 3, 6, and 10 of cardiac differentiation):
  a. Aspirate the old medium on the differentiation 12-well plates and wash the cells with DPBS once.
  b. Add 0.5 mL of Accutase to each well of the 12-well plate. Put the plate in a 37°C, 5% CO_2_ incubator for 5–10 min.
  c. Add 0.5 mL of mTeSR1 or FBS to each well. Collect all the cells into a 15-mL conical tube by gently pipetting.
  d. Centrifuge the pooled cells at 200 × *g* for 5 min at 20°C–25°C.
  e. Aspirate the supernatant and resuspend the cells in 100 μL of DPBS.
  f. Count the total cell number using an automated cell counter or a hemocytometer.
  g. Centrifuge the cells at 200 × *g* for 5 min at 20°C–25°C.
  h. Aspirate the supernatant and resuspend the cells in Blocking/Staining Solution A.
  i. Divide the cell solution into separate 1.5 mL tubes for unstained and single color-stained controls, and multicolor-stained samples.

***Note:*** The cell concentration (cell number per volume) of each tube for the single color-stained controls and multicolor-stained samples should be the same (e.g., 1–2 × 10^6^ cells per 100-200 μL of Blocking/Staining Solution A) (see TroubleshootingProblem 2).

11. Staining for cell surface markers:
  a. Do blocking of the cells in Blocking/Staining Solution A on the shaker at 4°C for 15 min.
  b. Centrifuge the cells at 200 × *g* for 5 min at 20°C–25°C.
  c. Aspirate the supernatant and resuspend the cells in 100–200 μL of the primary antibodies’ solution (Blocking/Staining Solution A + appropriately diluted primary antibodies for cell surface antigens; e.g., anti-platelet derived growth factor receptor-α [PDGFRα]) (Table 1).

**Table 1:** Antibodies to sort hESC-derived cardiac subpopulations via FACS

	Cell surface or intracellular antigen	Company	Catalog number	Dilution
**Primary antibody**				

Cardiac troponin T/TNNT2 FITC	intracellular	Abcam	ab105439	1:200
CD31 APCCY7	cell surface	BD Biosciences	563653	1:150
HCN4 PE	cell surface	StreeMarq Biosciences	SMC-320D-R-PE	1:200
ISL1	intracellular	DSHB	39.4D5	1:100
ISL1 PE	intracellular	BD Biosciences	562547	1:100
PDGFRα Alexa Fluor (AF) 647	cell surface	BD Biosciences	562798	1:100
SMMHC	intracellular	Biomedical Technologies	BT-562	1:20
OCT3/4	intracellular	Santa Cruz	sc-5279	1:50

	Targeted primary antibody	Company	Catalog number		Dilution
**Secondary antibody**					

Goat anti-mouse IgG2b secondary antibody, AF 647	ISL1 (39.4D5)	Thermo Fisher Scientific	A-21242	1:1,000	
Goat anti-rabbit IgG (H+L) secondary antibody, AF 405	SMMHC (BT-562)	Thermo Fisher Scientific	A-31556	1:500	
Goat anti-mouse IgG2b secondary antibody, AF 488	ISL1 (39.4D5), OCT3/4 (sc-5279)	Thermo Fisher Scientific	A-21141	1:1,000	

**Isotype control**					

Mouse IgG1 FITC	TNNT2 FITC (ab105439)	Abcam	Ab91356	1:20	
Mouse IgG1 APCCY7	CD31 APCCY7 (563653)	BD Biosciences	557873	1:150	
Mouse IgG1 PE	HCN4 PE (SMC-320D-R-PE)	Thermo Fisher Scientific	12-4714-42	1:20	
Mouse IgG2b	ISL1 (39.4D5)	Thermo Fisher Scientific	14-4732-82	1:100	
Mouse IgG1 PE	ISL1 PE (562547)	BD Biosciences	554680	1:50	
Mouse IgG2a AF 647	PDGFRα AF647 (562798)	BD Biosciences	557715	1:100	
Rabbit IgG	SMMHC (BT-562)	Thermo Fisher Scientific	02-6102	1:20	
Mouse IgG2b AF 488	OCT3/4 (sc-5279)	Santa Cruz	sc-3892	1:50
  d. Incubate the cells on the shaker at 4°C for 30–60 min.
  e. Centrifuge the cells at 200 × *g* for 5 min at 20°C–25°C and wash the cells with 300 μL of DPBS.
  f. Repeat step 11e.
  g. Centrifuge the cells at 200 × *g* for 5 min at 20°C–25°C and aspirate the supernatant.

12. After cell surface markers’ staining (step 11), one of the following steps (a, b, or c) should be adopted:
  a. If either staining for inner cell proteins or staining with a secondary antibody is not planned, resuspend the cells in 300 μL of FACS buffer and filter the cell solutions into a 5 mL FACS tube. Keep the FACS tubes on ice until detection. Go to step 18.
    ***Note:*** For live cell sorting, the cells should be stained with DAPI (4′,6-diamidino-2-phenylindole; a final concentration: 0.05–0.2 μg/mL) or PI (propidium iodide; a final concentration: 2–5 μg/mL) immediately before the FACS running, so that live intact cells can be gated as DAPI- or PI-negative populations.
  b. If the primary antibody used in step 11 is not conjugated with a fluorochrome, cells should be stained with a fluorochrome-conjugated secondary antibody (e.g., Alexa Fluor 405-, 488-, 555-, and/or 647-conjugated [Molecular Probes]; typically diluted at 1:500–1,000 in 500–1,000 μL of Blocking/Staining Solution A) specific to the appropriate species on the shaker at 4°C for 15 min. Thereafter, return to step 11e–g, and then follow either step 12a or 12c.
  c. If staining for inner cell proteins is concurrently planned, go to step 13.
    ***Note:*** In the case of the isolation of hESC-derived cardiac subpopulations described here, the step 12c should be adopted because the progenitor marker ISL1 is an inner cell protein and requires cell fixation for staining.

13. Fix the cells with a 4% paraformaldehyde (PFA) solution on the shaker at 20°C–25°C for 20–25 min. Wrap the cell tubes in aluminum foil.

***Note:*** After cells are once stained with fluorochrome-conjugated antibodies, protect the cells from the light at all times.

**CRITICAL:** Prepare all the buffers and solutions at and after fixation (in steps 13–18) to be supplemented with a RNase inhibitor (e.g., RNaseOUT Ribonuclease inhibitor [Thermo Fisher]) at a 1:100 ratio for effective RNA extraction from the fixed and FACS-sorted cells. This is also applied to PBS used for cell washing (see TroubleshootingProblem 3).

14. Centrifuge the cells at 400 × *g* for 5 min at 20°C–25°C. Aspirate the supernatant and resuspend the cells in 1 mL of Permeabilization solution.
15. Repeat step 14.
16. Centrifuge the cells at 400 × *g* for 5 min at 20°C–25°C. Aspirate the supernatant and resuspend the cells in 0.5 mL of Blocking/Staining Solution B.
17. Staining for inner cell proteins:
  a. Do blocking of the cells in Blocking/Staining Solution B on the shaker at 4°C for 15 min. Wrap the cell tubes in aluminum foil.
  b. Centrifuge the cells at 400 × *g* for 5 min at 20°C–25°C.
  c. Aspirate the supernatant and resuspend the cells in 100–200 μL of the primary antibodies’ solution (Blocking/Staining Solution B + appropriately diluted primary antibodies for inner cell proteins; e.g., anti-ISL1) (Table 1).
  d. Incubate the cells on the shaker at 4°C for 30–60 min.
  e. Centrifuge the cells at 400 × *g* for 5 min at 20°C–25°C and wash the cells with 300 μL of PBS.
  f. Repeat step 17e.
  g. Centrifuge the cells at 400 × *g* for 5 min at 20°C–25°C and aspirate the supernatant.
  h. Stain the cells with a fluorochrome-conjugated secondary antibody (typically diluted at 1:500–1,000 in 500–1,000 μL of Blocking/Staining Solution B) specific to the appropriate species on the shaker at 4°C for 15 min.
  i. Centrifuge the cells at 400 × *g* for 5 min at 20°C–25°C and wash the cells with 300 μL of PBS.
  j. Repeat step 17i.
  k. Centrifuge the cells at 400 × *g* for 5 min at 20°C–25°C. Aspirate the supernatant, and resuspend the cells in 300 μL of FACS buffer. Filter the cell solutions into a 5 mL FACS tube. Keep the FACS tubes on ice until detection.

**CRITICAL:** For the appropriate gating strategy at FACS, the following stained controls should be prepared in addition to unstained and single color-stained controls. Run and analyze all these controls with samples at the same time (see TroubleshootingProblem 2):

Isotype primary antibody (derived from the same species and immunoglobulin type)-stained control(s) (Table 1)

Only the secondary antibody-stained control(s)

Positively and negatively stained cell-type control(s) (For example, undifferentiated hESCs are often used for the latter.)

18. Analyze and sort the stained cells using a flow cytometer and cell sorter (e.g., FACSARIA III [BD Biosciences]) equipped with a 100 μm nozzle at 4°C temperature. Keep the flow rate between 800–1,200 events per second.
  a. Use the unstained and single color-stained controls to set appropriate PMT voltages and adjust compensation using a cell analyzing software (e.g., FACS Diva [BD Biosciences]).
  b. Gate single cells with the FSC-A versus SSC-A plot, the FSC-W versus FSC-H plot, and the SSC-W versus SSC-H plot to discard cell debris and doublets (Figure 2A). When performing live cell sorting, gate DAPI- or PI-negative cell populations (*see step 12a Note*).
  c. Create appropriate sorting gates for the samples through concurrently analyzing and referring to the isotype antibody-stained and the marker-positive or negative cell-type controls.
  d. Sort the ISL1^+^PDGFRα^+^ MCPs (day 3), the ISL1^+^Lin (i.e., TNNT2, HCN4, SMMHC, or PECAM1)^+^ cardiac intermediates (day 6), and the ISL1^-^Lin^+^ differentiated cardiac cells such as cardiomyocytes, pacemaker, smooth muscle, and endothelial cells (CM, PM, SMC, and EC; day 10) (Figures 2B–2D). Sort more than 10,000 (preferably, ≥20,000) fixed cells of each population into pre-coated 1.5 mL tubes containing 100 μL of PBS (see Troubleshooting Problem 3).
  e. Immediately after sorting, centrifuge the cells in 1.5 mL tubes at 400 × *g* for 5 min at 4°C and aspirate the supernatant. Go to step 19.
  f. Further analyze the flowcytometry data with the FlowJo software (Tree Star).

### RNA extraction from fixed, stained, and FACS-sorted cells

**Timing: 4–5 h**

Total RNA of the intracellular stained and FACS-sorted cells is extracted using an efficient protocol customized for fixed cells (Hrvatin et al., 2014) with minor modifications (i.e., incubation time length and temperature in protease digestion [step 19]).

**CRITICAL:** Before starting to work with RNA, clean the lab environments including a bench, pipettors, and other instruments with an RNase decontamination solution (e.g., RNaseZap solution [Ambion]).

19. Protease digestion: Resuspend the cell pellets (from step 18e) in 100 μL of Digestion Buffer with 4 μL of Protease (both supplied in the RecoverAll Total Nucleic Acid Isolation Kit [Ambion]; https://www.thermofisher.com/order/catalog/product/AM1975#/AM1975) in 1.5 mL tubes and incubate the mixture in heat blocks or a water bath at 50°C for 1 h.

**Pause point:** After protease digestion, the cell lysates can be snap-frozen and stored in a freezer at −80°C for 2–4 weeks until the following RNA isolation.

20. RNA isolation is conducted according to the manufacturer’s instructions (RecoverAll Total Nucleic Acid Isolation Kit [Ambion]), as described shortly below:
  a. Combine 120 μL of Isolation Additive (*supplied in the kit*) and 275 μL of 100% ethanol.
  b. Add 395 μL of the Isolation Additive/ethanol mixture into the cell lysate from step 19. Mix by gently pipetting.
  c. Pipet the cell lysate/ethanol mixture (from step 20b) onto a filter cartridge placed in a collection tube (*both supplied in the kit*).
  d. Centrifuge at 10,000 × *g* for 30 s to pass the mixture through the filter.
  e. Discard the flow-through and re-set the filter cartridge in the same collection tube.
  f. Add 700 μL of Wash 1 (*supplied in the kit*) to the filter cartridge.
  g. Centrifuge at 10,000 × *g* for 30 s. Discard the flow-through and re-set the filter cartridge in the same collection tube.
  h. Add 500 μL of Wash 2/3 (*supplied in the kit*) to the filter cartridge.
  i. Centrifuge at 10,000 × *g* for 30 s. Discard the flow-through and re-set the filter cartridge in the same collection tube.
  j. Centrifuge the assembly at 10,000 × *g* for an additional 30 s to remove residual fluid from the filter.
21. RNA purification with DNase digestion is conducted according to the manufacturer’s instructions (RecoverAll Total Nucleic Acid Isolation Kit [Ambion]), as described shortly below:
  a. Combine 4 μL of DNase, 6 μL of 10× DNase buffer, and 50 μL of nuclease-free water (*all supplied in the kit*).
  b. Add 60 μL of DNase mix (from step 21a) to the center of the filter cartridge (from step 20j). Cap the tube and incubate for 30 min at 20°C–25°C.
  c. Add 700 μL of Wash 1 to the filter cartridge. Incubate for 60 s at 20°C–25°C.
  d. Centrifuge at 10,000 × *g* for 30 s. Discard the flow-through and re-set the filter cartridge in the same collection tube.
  e. Add 500 μL of Wash 2/3 to the filter cartridge.
  f. Centrifuge at 10,000 × *g* for 30 s. Discard the flow-through and re-set the filter cartridge in the same collection tube.
  g. Repeat steps 21e and 21f.
  h. Centrifuge the assembly at 10,000 × *g* for 1 min to remove residual fluid from the filter.
  i. Transfer the filter cartridge to a fresh collection tube.
  j. Apply 20–60 μL of Elution Solution (*supplied in the kit*) or nuclease-free water at 20°C–25°C to the center of the filter cartridge, and close the cap.
  k. Place the sample at 20°C–25°C for 1 min.
  l. Centrifuge for 1 min at maximum speed to pass the mixture through the filter. The eluate contains the RNA.
22. The integrity and concentration of extracted RNAs are measured with the Agilent RNA 6000 Nano kit and the 2100 Bioanalyzer instrument (Agilent) according to the manufacturer’s instructions (https://www.agilent.com/en/product/automated-electrophoresis/bioanalyzer-systems/bioanalyzer-instrument).

***Note:*** The RNA can be stored in a freezer at −80°C for 2–4 weeks until cDNA library construction (from step 36 onward).

### Isolation of human embryonic/fetal heart-derived single cardiac cells

**Timing: 3–4 h**

**Figure 3:**
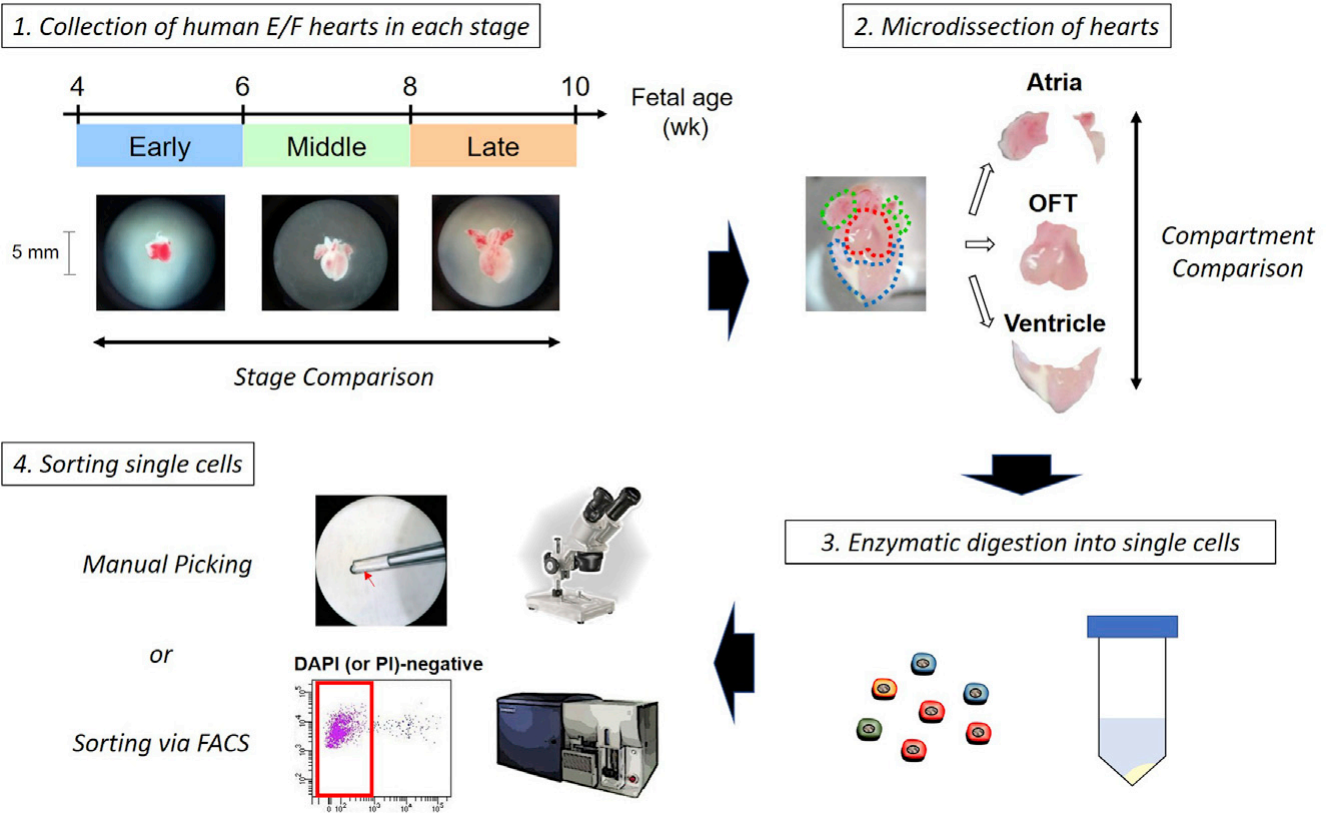
Workflow of the isolation of human embryonic/fetal heart-derived single cardiac cells E/F, embryonic/fetal; OFT, outflow tract.

This step enzymatically dissociates cardiac cells from each compartment of fresh human embryonic/fetal hearts (Figure 3).

**CRITICAL:** All the procedures here, except for digestion steps and centrifugation, should be performed under a UV-sterilized cabinet with laminar flow (Biosafety Level II) using standard aseptic precautions. Keep the experimental environment free from RNases and contaminated DNA.

23. Following surgery, quickly transfer human embryonic/fetal hearts (e.g., at 4.5 to 10 weeks of the gestation stages) into 35 mm dishes filled with 5 mL of DMEM/F12 on ice.
24. Under a microscope in the hood, dissect the hearts carefully into 3 compartments such as outflow tract, ventricle, and atria with removing fatty and connective tissues using fine small and spring scissors (straight type) and forceps (standard and 45° tips). Transfer the regions separately into different 35 mm dishes filled with 5 mL of the Heart-Primary solution on ice.
25. Cut each region into as small pieces as possible, using the scissors and forceps.
26. Transfer cut pieces of each region into 3 mL of the Collagenase A solution in a 15 mL tube, separately.

**CRITICAL:** Prepare fresh collagenase A and B solutions every time.

27. Pre-digestion: Place the tubes into a water bath at 37°C with shaking for 10 min.

***Note:*** The pre-digestion step removes red blood cells and cell debris from the samples.

28. Centrifuge the tubes at 200 × *g* for 3 min. Discard the supernatant carefully in each tube.
29. Add 3 mL of the Collagenase B solution into the 15 mL tubes. Gently tap the bottom of the tubes.
30. Digestion: Place the tubes into a water bath at 37°C with shaking for 25 min.

***Note:*** If the water bath does not have an automated shaking function, shake the tubes manually for 1 min every 5 min.

31. Transfer and pass the suspension including dissociated single cells through a 40-μm cell strainer into a new 50 mL tube filled with 1 mL of FBS for neutralization.

***Note:*** If there are still clearly visible residual tissues, add another 3 mL of the Collagenase B solution into the same 15 mL tube after transferring the supernatant, and repeat step 30. Finally, the dissociated cell suspensions from the first and second digestion steps can be combined (see TroubleshootingProblem 4).

32. Centrifuge the 50 mL tubes at 200 × *g* for 3 min. Discard the supernatant carefully.
33. Resuspend the cells in 200 μL of Cell Suspension Buffer (containing TrypLE Express Enzyme and DPBS at a 1:1 ratio) for manually picking single cells (Go to step 34), or 300 μL of FACS buffer for sorting single cells with FACS (Go to step 35).

34. Manual picking of single cells:
  a. Transfer the cell solutions (in Cell Suspension Buffer) into a new 35 mm dish.
  b. Using a micro capillary pipette under a microscope in the hood, pick single cells in the volume of 0.3 μL and transfer each single cell into a 0.2-mL thin-walled PCR tube containing 2 μL of Cell Lysis Buffer (i.e., nuclease-free water containing 0.2% Triton X-100 and 2 U/μL of a RNase inhibitor), 1 μL of 10 μM oligo-dT primer (5′-AAGCAGTGGTATCAACGCAGAGTACT_30_VN-3′) and 1 μL of 10 mM dNTP mix.
    ***Note:*** The 0.2-mL thin-walled PCR tubes containing 2 μL of Cell Lysis Buffer, 1 μL of 10 μM oligo-dT primer and 1 μL of 10 mM dNTP mix per one tube should be prepared before picking cells.
  c. After isolating, the single cells in the 0.2-mL thin-walled PCR tubes are snap-frozen on dry ices and stored in a freezer at −80°C for 2–4 weeks until cDNA library construction (from step 37 onward).
    **CRITICAL:** All the procedures from the heart digestion to picking single cells (steps 23–34) should be completed within 3–4 h, to prevent loss of the cell viabilities. Typically, 150–200 single cells from each region (i.e., outflow tract, ventricle, or atria) can be obtained during the time interval (see TroubleshootingProblem 5).

35. Single-cell sorting via FACS:
  a. Filter the cell solutions (in FACS buffer) into a 5 mL FACS tube. Keep the FACS tubes on ice until detection.
  b. Using a cell sorter (e.g., FACSARIA III) and following step 18b, DAPI- or PI-negative live cells are gated and sorted as one cell per well on 96- or 384-well plates where each well contains 2 μL of Cell Lysis Buffer, 1 μL of 10 μM oligo-dT primer and 1 μL of 10 mM dNTP mix.
    ***Note:*** The 96- or 384-well plates containing 2 μL of Cell Lysis Buffer, 1 μL of 10 μM oligo-dT primer and 1 μL of 10 mM dNTP mix in each well should be prepared in advance and can be stored at −20°C for 2–4 weeks. After the solutions in the plate are thawed on ice, centrifuge the plate at 200 × *g* for 30 s before sorting cells.
  c. After sorting, the 96- or 384-well plates are snap-frozen on dry ices and stored in a freezer at −80°C for 2–4 weeks until cDNA library construction (from step 37 onward).

### Construction of cDNA libraries with Smart-Seq2

**Timing: 10–12 h**

cDNA libraries of the hESC-derived cardiac bulk populations and the human embryonic/fetal heart-derived single cardiac cells are generated with the Smart-Seq2 method (Picelli et al., 2013) with minor modifications (i.e., we also apply Smart-Seq2 to bulk RNA samples when minor cell populations obtained with very low amounts of RNA due to a fewer cell number and cell fixation before RNA extraction are included). The generated cDNA libraries are ready for sequencing.

***Note:*** For bulk populations’ RNA samples (from step 22), go to step 36, and for single-cell lysates (from step 34 or 35), go to step 37 directly.

36. Transfer 100 pg of total RNA from bulk populations (from step 22) into a 0.2-mL thin-walled PCR tube, containing 1 μL of 10 μM oligo-dT primer and 1 μL of 10 mM dNTP mix. Add nuclease-free water to a total volume of 4.3 μL in each sample.
37. Denature: RNA solutions (from step 36) or single-cell lysates (from step 34 or 35) are denatured at 72°C for 3 min and immediately placed on ice afterward.
38. Add 5.7 μL of the reverse transcription (RT) mix, containing 0.5 μL of SuperScript II reverse transcriptase (200 U/μL) and 0.1 μL of 100 μM Template-switching oligo (5′-AAGCAGTGGTATCAACGCAGAGTACATrGrG+G-3′), to each sample (a final reaction volume of 10 μL). Mix the reaction by gently pipetting and spin down the samples at 600 × *g* for 15 s at 20°C–25°C.
39. Reverse transcription: Incubate the sample tubes in a thermal cycler as the following condition: 42°C for 90 min, 10 cycles of “50°C for 2 min and 42°C for 2 min,” and 70°C for 15 min. Set and keep the thermal cycler at 4°C after the reaction terminated.
40. Add 15 μL of the PCR pre-amplification mix, containing 12.5 μL of KAPA HiFi HotStart Ready Mix (2×, KAPA Biosystems) and 0.25 μL of 10 μM IS-PCR primer (5′-AAGCAGTGGTATCAACGCAGAGT-3′), to each sample (a final reaction volume of 25 μL). Mix the reaction by gently pipetting and spin down the samples at 600 × *g* for 15 s at 20°C–25°C.
41. PCR pre-amplification: Incubate the sample tubes in a thermal cycler as the following condition: 98°C for 3 min, 15 cycles (for bulk population RNA) or 18 cycles (for single cells) of “98°C for 20 s, 67°C for 15 s, and 72°C for 6 min,” and 72°C for 5 min. Set and keep the thermal cycler at 4°C after the reaction terminated.

**Pause point:** The PCR products can be stored in a freezer at −20°C for 1–2 months until the following procedures.

42. PCR purification: Purify the PCR products using AMPure XP beads (Beckman Coulter):
  a. Equilibrate AMPure XP beads at 20°C–25°C for 15 min. Then, vortex well.
  b. Add 25 μL of AMPure XP beads (1:1 ratio) to each sample from step 41. Mix the reaction by pipetting and transfer the solutions to a 96-well plate.
  c. Incubate the mixture for 8 min at 20°C–25°C.
  d. Place the 96-well plate on the magnetic stand (e.g., Magnetic Stand-96 [Ambion]) for 5 min.
  e. Discard the clear liquid carefully.
  f. Wash the beads with 200 μL of 80% ethanol solution. Incubate the samples for 30 s and then discard the ethanol.
  g. Repeat the washing (step 42f).
  h. Dry the beads by leaving the plate at 20°C–25°C for 5 min.
  i. Add 17.5 μL of nuclease-free water and resuspend the beads.
  j. Incubate the plate without the magnetic stand for 2 min.
  k. Place the plate on the magnetic stand for 2 min.
  l. Pipette 15 μL of the clear supernatant without disturbing the beads and transfer it to a 0.2-mL thin-walled PCR tube.
43. First quality check of the constructed cDNA library: Check the library size distribution using a High-Sensitivity DNA chip and a Bioanalyzer (Agilent).

**Figure 4:**
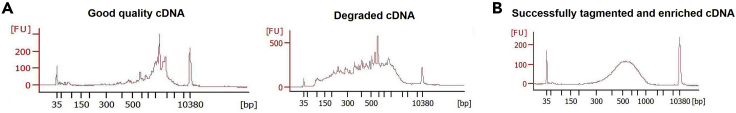
Examples of cDNA library profiles obtained by the Bioanalyzer system (A) Representative examples of good quality cDNA (left) and degraded cDNA (right) after the PCR pre-amplification. (B) Representative example of successfully tagmented and enriched cDNA after the final enrichment PCR.

***Note:*** The expected average library size should be around 1.5–2.0 kb with few short (<500 bp) fragments (Figure 4A).

44. Tagmentation reaction:
  a. Set up the tagmentation reaction solution, containing 500 pg of cDNA (from step 42l), 10 μL of a tagment DNA buffer (2×) and 5 μL of a tagment DNA enzyme mix (Nextera XT DNA Library Preparation kit [Illumina]), to a final reaction volume of 20 μL on ice. Mix the solution by pipetting.
  b. Perform the tagmentation reaction in a thermal cycler at 55°C for 5 min. Set and keep the thermal cycler at 4°C after the reaction terminated.
  c. To strip off the tagment enzyme, add 5 μL of NT buffer (Nextera XT DNA Library Preparation kit) to each sample. Mix the solution by pipetting.
  d. Incubate the mixture for 5 min at 20°C–25°C.
45. Final enrichment PCR:
  a. Prepare the enrichment PCR solution (a final reaction volume of 50 μL), containing 25 μL of tagmented DNA (from step 44d), 15 μL of Nextera PCR Master Mix, 5 μL of Index 1 primers (N7xx) and 5 μL of Index 2 primers (N5xx), using the Nextera XT DNA Library Preparation and Index kits (Illumina).
  b. Perform PCR in a thermal cycler as the following condition: 72°C for 3 min, 95°C for 30 s, 12 cycles of “95°C for 10 s, 55°C for 30 s, and 72°C for 30 s,” and 72°C for 5 min. Set and keep the thermal cycler at 4°C after the reaction terminated.
46. PCR purification: Repeat step 42 (a to l) but use 30 μL of AMPure XP beads (at a 0.6:1 ratio). Finally, transfer 15 μL of the clear supernatant into a new 1.5-mL polyallomer tube.
47. Quality check of the final cDNA library: Measure the concentration of each library by a Qubit High-Sensitivity DNA kit and a Qubit fluorometer (Invitrogen) according to the manufacturer’s instructions (https://www.thermofisher.com/se/en/home/industrial/spectroscopy-elemental-isotope-analysis/molecular-spectroscopy/fluorometers/qubit/qubit-fluorometer.html). Check the size distribution of the tagmented library using a High-Sensitivity DNA chip and a Bioanalyzer (Agilent).

***Note:*** The expected size of the tagmented library is around 300–900 bp (Figure 4B).

48. Library pooling:
  a. Calculate the molarity of the final library using the concentration obtained with the Qubit system and the average library size obtained with the Bioanalyzer (from step 47).
  b. Dilute each library to a final concentration of 4 nM. Pool equal nanomoles of each sample, confirming that none of them has the same combination of Index 1 (N7xx) and Index 2 (N5xx) adapters.
  c. Measure again the concentration and the library size distribution of the pooled libraries by the Qubit and Bioanalyzer systems. Adjust the concentration to 4 nM, if necessary.

***Note:*** After this step, the constructed cDNA library is ready for sequencing. Perform single-end or paired-end sequencing of the libraries using a next-generation sequencer (e.g., HiSeq 2500 [Illumina]; http://www.illumina.com/) according to the manufacturer’s and the sequence core facility’s instructions.

### Expected outcomes

**Figure 5:**
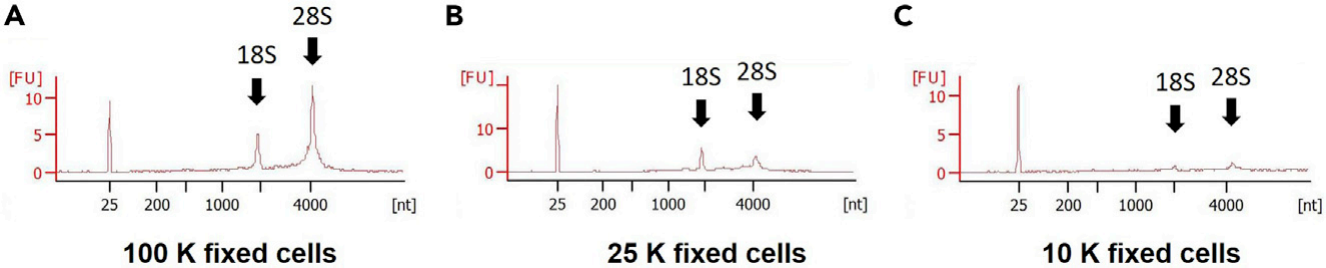
Examples of RNA profiles of fixed and FACS-sorted cells with different cell numbers Results obtained by the Bioanalyzer system. The RNA integrity number (RIN) scores: (A) 8.6, (B) 8.0, and (C) N/A.

By applying the cardiac differentiation protocol (Figure 1) and the multicolor cell separation approach with lineage-specific markers’ antibodies (Table 1), each of the stage-specific cardiac lineages, such as the ISL1^+^PDGFRα^+^ MCPs (day 3), the ISL1^+^Lin (i.e., TNNT2, HCN4, SMMHC, or PECAM1)^+^ cardiac intermediates (day 6), and the ISL1^−^Lin^+^ differentiated cardiac cells (CM, PM, SMC, and EC) (day 10) can be isolated via FACS (Figure 2). Under successful cardiac differentiation, while a great number of cells (e.g., approximately 1–2 million cells from one 12-well culture plate) of the cardiomyocyte lineage (ISL1^+^TNNT2^+^ [day 6] and ISL1^−^TNNT2^+^ [day 10]) can be sorted, around 10,000 cells in each of the other minor populations (i.e., pacemaker, smooth muscle, and endothelial lineages) can be sorted from one 12-well culture plate. Using the RNA extraction protocol described here, we usually observe the good quality of RNA profiles from more than 10,000 (preferably, ≥20,000) fixed and FACS-sorted cells in each population (Figure 5). With the heart digestion and isolation approach (Figure 3) in this protocol, followed by the cDNA library construction procedure with the Smart-Seq2, the good quality of cDNA library profiles can be obtained from 500–1,000 single cardiac cells derived from one human embryonic/fetal heart (Figure 4). At the end of this protocol, the population and single-cell libraries are ready for sequencing. After sequencing and getting raw sequenced FASTQ files, downstream bioinformatics analysis will allow us to uncover developmental cellular hierarchies and a molecular atlas of individually specified cells in more detail through combining both the population and single-cell transcriptomic data (Sahara et al., 2019). The approach described here could be applicable to the studies investigating not only cardiogenesis but also other organs’ development in human embryogenesis.

## Limitations

RNA degradation often happens, especially in the lowest amount of RNA samples derived from single cells and/or fixed and FACS-sorted rare cell populations. This hampers construction of the valid cDNA libraries, which is necessary for proper RNA sequencing and transcriptomic analysis. Therefore, the study described in this protocol requires to repeat the experiments many times, to optimize the conditions in each step, and to submit a great number of samples from the precious materials such as human embryonic/fetal tissues to sequencing, which means that these attempts are highly expensive and time consuming.

Although the cardiac differentiation protocol based on the Wnt signaling modulation (Figure 1) is efficient to differentiate hESCs toward the cardiac lineage (cardiac progenitors, intermediates, and cardiomyocytes), it is important to keep in mind that this approach is still somewhat artificial and may not reflect the *in vivo* authentic cardiac development precisely.

The limited cell numbers in the single-cell analysis by the Smart-Seq2 method may still bias the results. Especially, it might be difficult to detect rare and/or sensitive cell types and genes among the analyzed samples. Most recently, the Smart-Seq3 method has been reported to increase sensitivity for detecting transcripts per cell, compared to Smart-Seq2 (Hagemann-Jenson et al., 2020). We envision that our protocols described here could be adapted to Smart-Seq3 as well without major procedural changes, which may overcome those shortcomings.

## Troubleshooting

### Problem 1

The ESCs do not differentiate efficiently into cardiomyocytes.

### Potential solution

First, in every 12-well plates of cardiac differentiation, leave 1–2 wells without harvesting cells and continue the differentiation culture in those wells until 12–14 days of differentiation, in order to ensure that the cells on the plate could differentiate properly into beating cardiomyocytes by observing robust and broad spontaneous contractions from day 10–12 onward.

When not well differentiated, check if the hESCs maintained and used for differentiation entirely express pluripotency markers (e.g., Oct3/4) via immunocytochemistry or flow cytometry, and verify that the hESCs show a normal karyotype using standard G-band karyotype analysis (Tidball et al., 2017; Sun et al., 2020). Optimize the starting cell density and the concentration of CHIR99021 (or CHIR98014) for the differentiation, since these parameters are varied and dependent on the cultured cell lines, passage numbers, and other conditions. On day 1, 3, and 5 in cardiac differentiation, change the medium precisely 24, 72, and 96 h after the addition of CHIR99021 (or CHIR98014) on day 0. Use the freshly prepared mediums and chemicals (e.g., Y-27632, CHIR99021, and IWP2).

### Problem 2

The FACS analysis shows that some cell population is under-stained OR over-stained.

### Potential solution

Prepare the FACS tubes of single color-stained controls and multicolor-stained samples, all of which contain the stained cells with the same cell number per volume (e.g., 1–2 × 10^6^ cells per 100–200 μL of solution). For the appropriate gating, various staining controls, such as the isotype primary antibody-stained control, only the secondary antibody-stained control, and positively and negatively stained cell-type controls should be prepared and analyzed at the same time in addition to unstained and single color-stained controls. Optimize the dilution ratio of the antibodies in each case.

### Problem 3

The RNA profiles of the fixed and FACS-sorted cell populations show low quality (RNA degradation).

### Potential solution

Check if all the buffers and solutions at and after fixation (in steps 13–18) including PBS used for cell washing are supplemented with a RNase inhibitor at a 1:100 ratio. Sort as many fixed cells as possible (preferably, ≥20,000). For this, prepare ≥2–3 of the 12-well plates for cardiac differentiation at the same time, and collect the cells from those plates all together on the designated days.

### Problem 4

The human embryonic/fetal heart tissues are not well digested, and the number of the picked or sorted single cardiac cells is low.

### Potential solution

Cut the heart regions into as small pieces as possible under a microscope, using fine scissors and forceps. Prepare the collagenase A and B solutions freshly every time (Do not stock them into the freezer). The incubation time with the collagenase B solution can be extended by 30–40 min. If needed, repeat the digestion step using the collagenase B solution (step 30) and combine all the dissociated cell suspensions in the end.

### Problem 5

The constructed cDNA library profiles of the single cardiac cells show low quality (RNA degradation, etc.).

### Potential solution

Perform all the single-cell experiments in a specified area that is thoroughly cleaned and decontaminated from RNases and DNA. Complete the steps of the heart digestion and isolation of the single cardiac cells by manual picking or FACS sorting in the shortest time (within 3–4 h, at longest). After picking or sorting, immediately put the cells in cell lysis buffer onto dry ices and store at −80°C until cDNA library construction. Work fast and smoothly with the freshly prepared reaction solutions at each step of the Smart-seq2 (step 37 onward).

## Resource availability

### Lead contact

Further information and requests for resources and reagents should be directed to and will be fulfilled by the lead contact, Makoto Sahara (makoto.sahara@ki.se).

### Materials availability

This protocol does not generate new unique reagents.

### Data and code availability

Population RNA-seq data are available in the ArrayExpress database at EMBL-EBI (accession number: E-MTAB-7537; http://www.ebi.ac.uk/arrayexpress). Single-cell RNA-seq data are available in the Sequence Read Archive (SRA) (accession number: PRJNA510181; www.ncbi.nlm.nih.gov/sra/).

## References

[bib1] Burridge P.W., Matsa E., Shukla P., Lin Z.C., Churko J.M., Ebert A.D., Lan F., Diecke S., Huber B., Mordwinkin N.M. (2014). Chemically defined generation of human cardiomyocytes. Nat. Methods.

[bib2] Hagemann-Jensen M., Ziegenhain C., Chen P., Ramsköld D., Hendriks G.J., Larsson A.J.M., Faridani O.R., Sandberg R. (2020). Single-cell RNA counting at allele and isoform resolution using Smart-seq3. Nat. Biotechnol..

[bib3] Hrvatin S., Deng F., O'Donnell C.W., Gifford D.K., Melton D.A. (2014). MARIS: method for analyzing RNA following intracellular sorting. PLoS One.

[bib4] Lian X., Zhang J., Azarin S.M., Zhu K., Hazeltine L.B., Bao X., Hsiao C., Kamp T.J., Palecek S.P. (2013). Directed cardiomyocyte differentiation from human pluripotent stem cells by modulating Wnt/beta-catenin signaling under fully defined conditions. Nat. Protoc..

[bib5] Picelli S., Björklund Å.K., Faridani O.R., Sagasser S., Winberg G., Sandberg R. (2013). Smart-seq2 for sensitive full-length transcriptome profiling in single cells. Nat. Methods.

[bib6] Sahara M., Santoro F., Sohlmér J., Zhou C., Witman N., Leung C.Y., Mononen M., Bylund K., Gruber P., Chien K.R. (2019). Population and single-cell analysis of human cardiogenesis reveals unique LGR5 ventricular progenitors in embryonic outflow tract. Dev. Cell.

[bib7] Sun L., Li J., Li E., Niu S., Qin Z., Zhi Q., Zhao J., Xiong H., Li Y., Jian L. (2020). CRISPR/Cas9 mediated establishment of a human CSRP3 compound heterozygous knockout hESC line to model cardiomyopathy and heart failure. Stem Cell Res..

[bib8] Tidball A.M., Dang L.T., Glenn T.W., Kilbane E.G., Klarr D.J., Margolis J.L., Uhler M.D., Parent J.M. (2017). Rapid generation of human genetic loss-of-function iPSC lines by simultaneous reprogramming and gene editing. Stem Cell Reports.

